# Mimicking Gastric Cancer Collagen Reorganization with Decellularized ECM-Based Scaffolds

**DOI:** 10.3390/biology14081067

**Published:** 2025-08-16

**Authors:** Néstor Corro, Sebastián Alarcón, Ángel Astroza, Roxana González-Stegmaier, Carolina Añazco

**Affiliations:** 1 Nutritional Biochemistry Laboratory, School of Nutrition and Dietetics, Faculty of Rehabilitation and Quality of Life Sciences, Universidad San Sebastián, Valdivia 5091000, Chile; ncorroa@docente.uss.cl; 2 Departamento de Ciencias Biológicas y Químicas, Facultad de Ciencias, Universidad San Sebastián, Sede Valdivia, Valdivia 5091000, Chile; angel.astroza@uss.cl; 3 Cancer Biology Laboratory, Facultad de Medicina, Universidad San Sebastián, Sede Concepción, Campus Tres Pascualas, Concepción 4080871, Chile; sebastian.alarcon@uss.cl; 4 Translational Medicine Laboratory, Instituto Oncológico Fundación Arturo López Pérez, Santiago 8320000, Chile; roxana.gonzalez@falp.org

**Keywords:** biomaterials, cancer-associated fibroblasts, cancer progression, collagen cross-linking, decellularized tissues, extracellular matrix, tumor microenvironment, gastric cancer, lysyl oxidase, lysyl hydroxylase, matrix metalloproteinases

## Abstract

The tumor microenvironment (TME) has a substantial impact on the progression of gastric cancer. Collagen, the most abundant protein in the extracellular matrix (ECM), forms a dense physical barrier that regulates anti-tumor immunity in the TME. It is a significant regulator of the signaling pathways of cancer cells, which are responsible for migration, proliferation, and metabolism. ECM proteins, particularly remodeling enzymes and collagens, can be modified to increase stiffness and alter the mechanical properties of the stroma. This, in turn, increases the invasive potential of tumor cells and resistance to immunotherapy. Given the dynamic nature of collagen, novel therapeutic strategies have emerged that target both collagen biosynthesis and degradation, processes that are essential for addressing ECM stiffening. This review delineates the upregulation of the expression and deposition of collagen, as well as the biological functions, assembly, and reorganization that contribute to the dissemination of this aggressive malignancy. Furthermore, the review emphasizes the importance of creating 3D in vitro models that incorporate innovative biomaterials that avoid the difficulties of traditional 2D culture in accurately simulating real-world conditions that effectively replicate the distinctive collagen microenvironment. Ultimately, it investigates the use of decellularized ECM-derived biomaterials as tumor models that are designed to precisely replicate the mechanisms associated with the progression of stomach cancer.

## Simple Summary

The collagen reorganization in the TME strongly impacts gastric cancer progression. Collagen, the most abundant ECM protein, produces a robust physical barrier that regulates TME anti-tumor immunity and cancer cell migration, proliferation, and metabolic signaling. Enzyme and collagen remodeling increase stiffness and mechanical properties. Tumor cells become more invasive and immunoresistant. As a result of its dynamic nature, ECM stiffening requires special treatment methods that target collagen creation and degradation. This review describes the changes in collagen expression and deposition, biological activities, assembly, and rearrangement which contribute to this aggressive malignancy. Additionally, 3D in vitro models with novel biomaterials are needed to effectively recreate real-world circumstances and the collagen microenvironment. Decellularized ECM-derived scaffolds could recreate stomach cancer growth as tumor models.

## 1. Introduction

Gastric cancer is the fifth most common type of cancer, with a higher incidence in Eastern Asia, Eastern Europe, and South America relative to other regions [1,2]. Despite the fact that the disease’s impact is decreasing as a result of enhanced nutrition, food preservation, prevention, earlier diagnosis, and treatment, it continues to have a poor prognosis, with metastasis being the main cause of gastric cancer mortality [3]. Gastric cancer is described as a multifactorial disease, with genetics, the environment, and nutrition playing key roles in the development of this disease [4].

Most patients are diagnosed at an advanced stage of gastric cancer, which affects their prognosis and access to treatment [2,3,5]. It has been shown that gastric cancer diagnosed in the early stages has a good prognosis, with a 5-year survival rate greater than 95%, while advanced or metastatic gastric cancer has a poor prognosis and low survival rates, with a 5-year survival rate of less than 20% [5,6]. To resolve this issue, it is essential to investigate novel approaches to enhance the comprehension and regulation of cellular processes like those in vivo and to advance drug screening for the development of therapeutic strategies for gastric cancer, emphasizing detection and new treatment alternatives for patients with primary gastric cancer that avoid metastasis.

**Figure 1:**
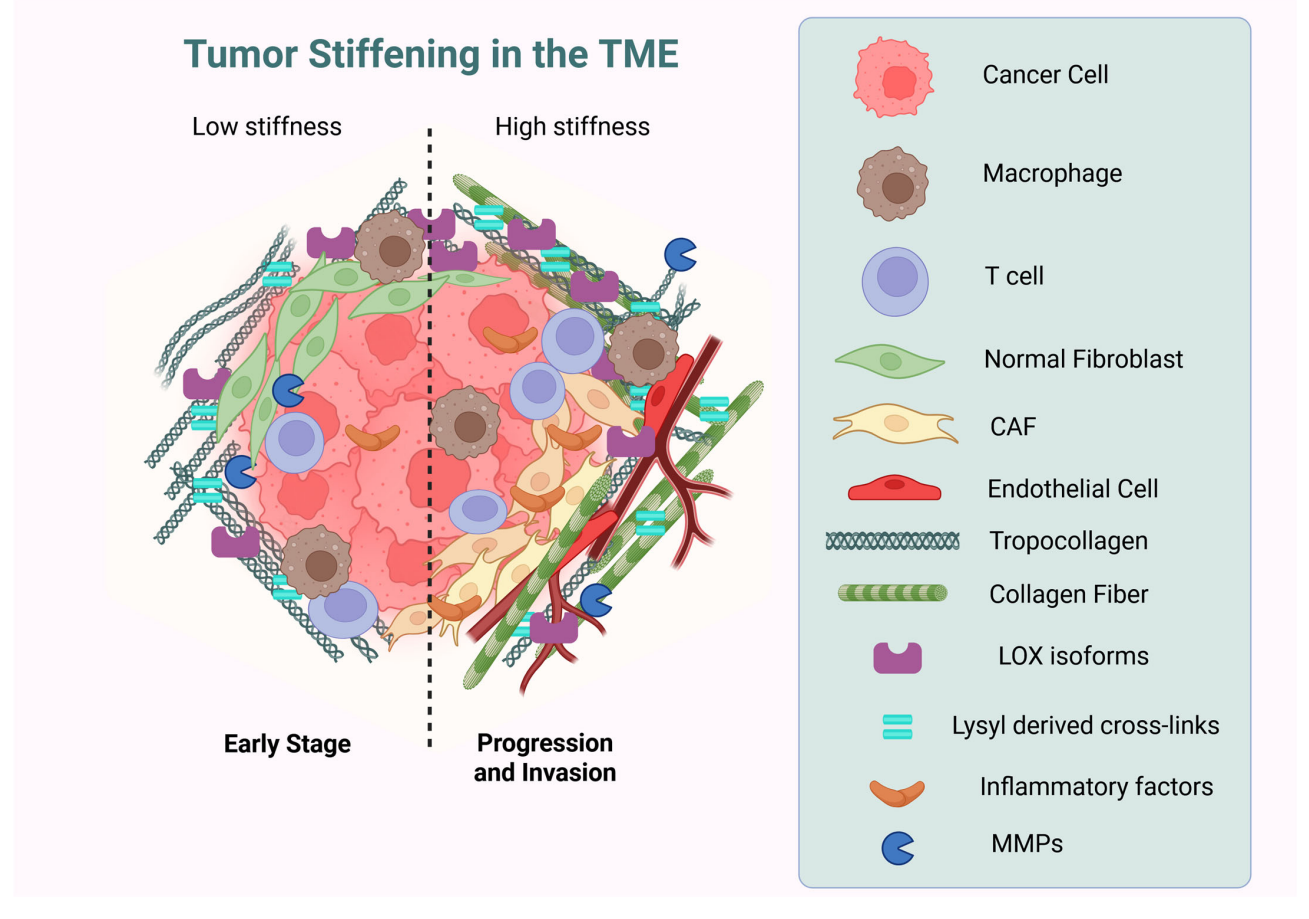
Cancer Cells and Tumor Microenvironment. The tumor microenvironment is crucial in inducing tumor invasion and metastasis due to the stiffness of the ECM due to the excessive collagen cross-linking by LOX systems, which induce several cellular processes such as proliferation, adhesion, and invasion, among others. LOX: lysyl oxidase; MMPs: matrix metalloproteinases. The figure was created with the license of BioRender.

Cancer invasion and metastasis result from interactions between cancer cells, immune cells, endothelial cells, fibroblasts, and the extracellular matrix (Figure 1) [7,8]. It has been reported that the deregulation of extracellular matrix (ECM) proteins has been observed in numerous types of cancer [9]. In this context, various pieces of evidence indicate that the ECM plays a central role in the formation of the tumor microenvironment (TME), and the alterations to the composition of this complex structure could play a significant role in the effects on chemotherapy and immunotherapy because it acts as a physical barrier to anti-cancer drugs [9,10,11]. In particular, the most abundant ECM protein, collagen, creates a dense physical barrier that impedes immune cell infiltration and influences anti-tumor immunity. Thus, it is essential to develop innovative biomaterials that effectively replicate the unique collagen microenvironment, as this has become a promising therapeutic target for addressing immunotherapy resistance.

According to numerous studies, approximately 95% of phase I anti-cancer medications are never released on the market. Although the causes of this rate of rejection are multifaceted, it is likely that it is the result of inefficient testing of potential treatment candidates. Drug evaluations often use cancer cells’ 2D in vitro models, which cannot replicate the complex 3D architecture and cellular content of the native TME. Oversimplification leads to excessive phenotypes and gene expression in cancer cells, resulting in non-physiological responses to medications. Animal models, including mice, pigs, and primates, although ethically controversial and expensive, do not accurately mimic human physiology, particularly the immune system within the TME. Additionally, they are incompatible with high-throughput production, which is essential for drug discovery and screening processes. To address this challenge, a new paradigm in tumor modeling has evolved in the last decade, utilizing discoveries in tissue engineering, materials science, nanotechnology, oncology, and pharmacology to enhance cancer therapies using 3D in vitro tumor models.

Innovative pre-clinical tumor models are revolutionizing drug discovery, testing, and therapy by replicating in vivo hierarchies, biological content, and complex dynamics in a controlled environment. Accurate information about tumor origins helps researchers understand growth, invasion, metastasis, and assess treatments before patient trials [12].

It has been unequivocally established that extracellular matrix (ECM) stiffening within the tumor microenvironment (TME) substantially contributes to enhanced cellular proliferation, metabolic reprogramming, cell adhesion, invasion, and metastasis, all of which are pivotal factors underlying the poor prognosis associated with gastric cancer [9,13,14]. Thus, comprehending the role of particular ECM components, like collagens, in the modulation of invasion and metastasis has heightened interest in creating biomaterial-based biomimetic ECM models to replicate essential tumor features [15]. Investigating the impact of collagen cross-linking and assembly in the TME is crucial for discovering new potential treatment targets for the pharmaceutical industry to prevent the progression of primary gastric cancer to advanced stages. Thus, decellularized extracellular matrices (dECM) have emerged as potential in vitro 3D tumor models, with recent advancements in processing and application positioning them as the best biomaterials for digestive system cancer research and the pharmaceutical sector.

This article provides a comprehensive examination of collagen subtypes and their post-translational alterations in the ECM of the gastric tumor microenvironment concerning gastric cancer development. Furthermore, we examine the essential elements of tumor stroma and their influence on tumor cell proliferation and invasion, focusing specifically on changes in collagen expression, deposition, rearrangement, and assembly. We also investigate alterations in the expression levels and activity of extracellular matrix-regulating enzymes in relation to metastatic gastric cancer. Recent studies of immunotherapy trials indicate that elevated collagen density and specific collagen gene signatures serve as credible predictors of inadequate responses to checkpoint inhibitors across various cancer types. To address immunotherapy resistance, it is essential to develop strategies that replenish or diminish the collagenous extracellular matrix. Ultimately, it examines the utilization of dECM-derived biomaterials as tumor models intended to accurately mimic the mechanisms associated with stomach cancer progression to explore the responses to pharmacological interventions aimed at the synthesis and degradation of collagen.

## 2. Gastric Cancer Overview

Gastric cancer is an aggressive solid tumor malignancy, and it ranks as the fifth most prevalent cancer globally, with an age-standardized incidence rate of 9.1 per 100,000 person-years, accounting for approximately 968,000 new cases in 2022 (constituting 4.9% of all cancer diagnoses). In addition, the age-standardized mortality rate was 6.1 per 100,000 people-years, making this cancer the fifth deadliest cancer. Gastric cancer was responsible for around 660,000 deaths, which accounted for 6.8% of all cancer-related deaths worldwide, as reported by the GLOBOCAN database [1,2,5]. Incidence rates of gastric cancer are twice as high in men compared to women [2]. In men, gastric cancer is the most diagnosed cancer and the primary cause of cancer mortality in various South-Central Asian countries [2]. Incidence rates of gastric cancer are highest in Eastern Asia, particularly in Mongolia for both sexes, and in Eastern Europe, whereas rates in Northern America, Northern Europe, and the African continent are generally low [1,2].

The Lauren classification system has been widely utilized for many years to classify gastric adenocarcinoma histologically into the well-differentiated intestinal subtype and the poorly differentiated diffuse subtype [16,17,18,19,20]. This classification system categorizes gastric adenocarcinoma into intestinal, diffuse, and indeterminate subtypes [21]. The intestinal type is believed to be primarily caused by environmental (exogenous) factors, while the diffuse type is thought to be due to hereditary and genetic (endogenous) factors [21,22,23].

Intestinal-type gastric cancer is commonly associated with *Helicobacter pylori* infection, which originates from chronic inflammation and progresses through atrophic gastritis, intestinal metaplasia, dysplasia, and finally gastric adenocarcinoma [24]. This subtype exhibits a higher prevalence among elderly individuals, particularly males, and is more common in high-risk regions [25]. From the histological perspective, the intestinal type is characterized by the presence of cohesive malignant epithelial cells and differentiated glandular cells that infiltrate the tissue in a heterogeneous manner, while the diffuse subtype contains poorly cohesive, highly undifferentiated tumor cells that can invade the entire stomach wall; signet ring cells and mucin can also be found dispersed throughout the stroma [18].

The diffuse type occurs in younger people, with an equal proportion of men and women, and is more common in areas of low risk [21]. The diffuse type, macroscopically, presents diffuse infiltration of tumorous tissue in the submucosa and muscularis propria layers of the stomach, with cells infiltrating the stomach wall, producing generalized thickening and rigidity. Microscopically, it presents stromal hypertrophy and hyperplasia with poorly differentiated adenocarcinoma and signet ring cell infiltration. Scirrhous gastric carcinoma, or diffusely infiltrative carcinoma, is associated with a poor prognosis attributed to its rapid infiltrative invasion and high rates of peritoneal dissemination [26]. Additionally, the diffuse type is molecularly identified by somatic or hereditary mutations of CDH1 gene, which codes for E-cadherin, a cell junction protein [27].

In addition to the intestinal and diffuse types, gastric cancer can be categorized into a mixed subtype, which histologically comprises tumors with both intestinal and diffuse components [21]. A study that included 814 gastric carcinomas, of which 415 were intestinal-type (51.0%), 221 were diffuse-type (27.1%), and 178 were mixed-type (21.9%) showed that mixed-type carcinomas exhibit more aggressive characteristics compared to intestinal and diffuse types, including larger size, deeper invasion, more frequent local invasion, and higher rates of lymph node metastasis [28].

The prognosis of patients diagnosed with gastric cancer is directly related to the size and degree of invasion into the inner layers of the stomach, which in turn determines the most appropriate treatment for each case. The TNM staging system (tumor, node, metastasis, with stages ranging from 0 to IV) created by the American Joint Committee on Cancer (AJCC) is used for the histopathological and clinical staging of gastric cancer, ranging from stage 0 to IV. While cases staged as localized disease (stage 0 to I) and advanced localized disease (stages II to III) can be treated with surgery with or without adjuvant treatments (chemotherapy, radiotherapy), cases of disseminated disease (stage IV) are only treated palliatively due to the low survival rate at this stage. The utilization of molecular markers that complement existing morphological and histopathological approaches can subsequently enhance the understanding of the pathogenesis and clinical significance of collagen assembly of stromal cells or cancer-associated fibroblasts and the tumor microenvironment of metastatic gastric cancer.

Histologically, gastric cancer has variable quantities of stroma, and the stroma has a significant impact on the growth and spread of the tumor. Studies have also demonstrated the significance of the tumor stroma in gastric cancer progression, which comprises various types of non-malignant cells, including endothelial cells, cancer-associated fibroblasts (CAFs), lymphocytes, macrophages, and non-cellular constituents, such as growth factors, enzymes, metabolites, and the ECM. It is well established that the interaction between the ECM and resident cells is critical for adequate normal gastric epithelial function.

Most of the research assessing the involvement of the tumor stroma in the microenvironment in gastric cancer has primarily concentrated on stromal cells, with little to no attention being paid to ECM components such as fibrillar collagens and their reorganization and assembly. Due to their extremely poor prognosis, scirrhous gastric cancers, a unique type of gastric cancer marked by extensive stromal fibrosis and collagen assembly, are typically used as an example to interpret the promotional role of collagen assembly in the progression of gastric cancer [29,30,31].

A comparative gene expression profile study between diffuse and intestinal types of gastric cancer revealed that the diffuse type exhibited changes in the expression of genes associated with ECM components, whereas the profile of the intestinal type indicated alterations in pathways related to cell growth and the cell cycle. Interestingly, increased rigidity of the ECM is also a hallmark of advanced diffuse-type gastric cancer, which can lead to a condition called linitis plastica characterized by diffuse infiltration of cancer cells throughout the stomach wall, leading to a marked loss of gastric distensibility and flexibility in the stomach [20,32]. This is a condition not usually associated with intestinal-type gastric cancer.

## 3. Collagens in Gastric Cancer: Expression, Deposition, Assembly, and Reorganization

To date, 28 collagen proteins (I–XXVIII) encoded by 44 genes have been identified [33]. Collagen is the most abundant structural component of the ECM and contributes significantly to tissue integrity and functionality, providing 3D scaffolding essential for biomechanical properties, cellular maintenance, and tissue architecture [34]. Tropocollagen, a physiological unit, consists of three polypeptide chains coiled into a right-handed triple helix. Monomers self-assemble into staggered arrays, creating collagen fibrils. Covalent cross-linking stabilizes and strengthens these fibrils. This porous, flexible matrix maintains cell quiescence and allows the easy flow of nutrients and immune cells [35].

In normal conditions, studies show that collagen IV is differentially distributed in human gastrointestinal tissues. The α1 (IV), α2 (IV), α5 (IV), and α6 (IV) collagen chains are expressed in the subepithelial basement membrane of all tissues, whereas α3 (IV) and α4 (IV) chains are expressed in specific regions of the gastric and intestinal epithelium in specific regions [36], suggesting that these chains play a protective role against the chemical and physical stress to which luminal epithelium is exposed.

In cancer, collagen fibers are the most prominent ECM component that undergoes dynamic changes in expression, organization, cross-linking, and density, impacting cancer initiation, progression, invasion, and metastasis [37,38]. The collagen fibers in the TME exhibit an increased diameter, enhanced density, and extensive cross-linking, resulting in the creation of a unique tumor ECM that is both highly dense and rigid. Furthermore, collagen engages with tumor and immune cells through the release of inflammatory factors, the exposure of binding sites, and the recognition of collagen receptors (Figure 1). It is well known that the dense collagen matrix, via integrins, enhances the interactions between cells and the extracellular matrix, thereby modulating the proliferation of stomach cancer cell lines [9].

Alterations in cancer cells trigger a series of events that lead to an increase in the rigidity of the ECM through collagen cross-linking, modifications in cell-–cell adhesion, and the activation of various cellular signaling cascades that promote tumor growth and dissemination [10,39]. Numerous studies demonstrate the variations in protein expression levels of different collagens between healthy tissue and stomach cancer samples, in addition to the association with increased collagen deposition and dynamic reorganization [40].

### 3.1. Upregulation of Collagens Expression and Deposition in Gastric Cancer

Transcriptomic analyses have shown that several collagen genes, including COL1A1, COL1A2, COL3A1, COL5A1, COL5A2, COL6A2, and COL6A3, are overexpressed during tumor growth from the T1 to T2 stages. The strong correlation in mRNA expression among these genes suggests that the targeted suppression of a single gene could undermine the synergistic effect of numerous genes. Consequently, these strongly associated genes may serve as prospective treatment targets in gastric cancer (Table 1) [41].

Previous studies have observed that changes in collagen within the tumor microenvironment are linked to the spread of cancer and its prognosis [42,43,44,45]. Moreover, the contribution of collagen at the interface between the tumor and stroma has been linked to the enhanced invasiveness of cancer cells [43,46]. A study in gastric cancer showed that those with a high collagen signature had a significantly higher 3-year cumulative rate of peritoneal metastasis compared to individuals with a low collagen signature (58.57% vs. 29.69%) [44].

Evidence shows that collagen type XII α1 chain (COL12α1) and type X α1 chain (COL10α1) expression is notably upregulated in gastric cancer. Jiang et al. (2019) analyzed protein levels of COL12α1 in a cohort of 200 GC samples and 184 benign tissues through IHC. COL12α1 was significantly overexpressed only in gastric cancer samples, correlated with tumor invasiveness, metastasis, and advanced clinical stage (TMN III + IV, *p*-value 0.01), suggesting an association with poor overall survival [47]. Similarly, Necula et al. (2020) analyzed COL10α1 expression in 49 GC tissue samples and plasma from patients and found higher levels in tumors versus adjacent benign tissue (*p*-value 0.05), and in patient plasma compared to 10 cancer-free controls (*p*-value 0.0029), reinforcing its prognostic relevance [48]. Another study developed by Sun et al. 2023 [41] studied the correlation between expression patterns of collagen genes and GC progression through TMN stages using RNAseq from biopsies allocated on the TCGA database. TCGA-based transcriptomic analyses revealed that COL10α1 and COL11α1 expression had the highest fold changes, correlating with the T stage, while COL4α5 mRNA levels were significantly related to poor overall survival (*p*-value 0.027). The authors suggest that the T staging reflects molecular ECM changes, noting that several collagen genes are differentially overexpressed between the T1 and T2 stages, when the tumor begins to invade the muscularis propria, highlighting their potential as early gastric cancer biomarkers [41]. Furthermore, a multiple bioinformatics analysis of 148 gastric cancer samples revealed that high expression of COL5α2 plays a key role in the prognosis of gastric cancer. According to the authors, high expression of COL5α2 and this protein could be potential biomarkers for this type of cancer [49] and has been associated with grades and stages of gastric cancer, impacting the survival rate of patients [50].

Studies show that the arrangement of collagen fibers in the tumor correlates with the degree of invasiveness it presents. Zhang et al. [51] determined that HAPLN1 (hyaluronan and proteoglycan link protein 1) was the most differentially up-regulated gene in CAFs obtained from 155 GC samples, through bioinformatics analysis, compared to normal fibroblasts (*p*-value 7.81 × 10^−6^). Furthermore, IHC analysis of tissue samples from the same cohort revealed a high correlation between HALPN1 gene expression and advanced tumor stage (TMN III–IV; *p*-value < 0.001). Xenograft models derived from these CAFs and gastric cancer cell lines in nude mice demonstrated that the reduction in density, width, and length of collagen, as well as the increase in the alignment of collagen fibers, is involved in greater invasiveness of the tumor. HALPN1 is a collagen crosslinker, and as the authors suggest, when it is overexpressed by CAFs, it could alter collagen cross-linking and turnover, reducing ECM contractility [51].

In addition, it has been shown that the collagen components in the stroma are predominantly synthesized by activated fibroblasts, known as myofibroblasts, which specifically express α-SMA. Immunohistochemistry analysis identified α-SMA-positive myofibroblasts in the stroma of both gastric cancer and non-neoplastic mucosa, revealing a greater prevalence in gastric cancer tissues. The authors concluded that a greater number of collagen-producing myofibroblasts may significantly contribute to the increased collagen deposition observed in gastric cancer [29]. Overall, these findings suggest that EMC remodeling is critical during gastric cancer progression, and it is driven primarily by CAFs and myofibroblasts through changes in collagen expression patterns. Although a detailed analysis of cancer-associated fibroblast (CAF) biology is beyond and detailed classification of CAF subtypes is beyond the scope of this review, cell-to-cell interaction and signaling pathways have been provided elsewhere [52], it is important to acknowledge their central role in modulating collagen deposition and architecture.

CAFs are heterogeneous in origin and function; their activation is primarily driven by paracrine signals from tumor cells, notably TGF-β1, IL-1β, PDGF, and Hedgehog ligands. These stimuli initiate transcriptional programs that define distinct CAF subsets. TGF-β1, through canonical SMAD2/3 signaling, induces a myofibroblast-like phenotype characterized by high expression of fibrillar collagens such as *COL1A1*, *COL3A1*, and *COL11A1* [53,54,55,56], promoting desmoplasia and matrix stiffening in gastric cancer, which are predictive of poor prognosis and enriched in CAF-high tumors [57]. Beyond collagen synthesis, CAFs in gastric cancer are key producers of matrix-modifying enzymes—including LOX and MMPs—that drive ECM cross-linking and focal degradation. LOX overexpression by CAFs correlates with increased tumor cell proliferation, migration, and invasion in gastric cancer tissues [58]. Similarly, high CAF infiltration correlates with increased MMP activity, promoting invasion and metastasis [59,60,61]. This suggests that CAF-derived collagens can be structurally altered through enhanced expression of ECM-modifying enzymes like LOX, PLOD2, and MMPs, resulting in a matrix more permissive to invasion and metastasis. Recent single-cell analyses of gastric cancer highlight COL11A1-positive fibroblasts as a tumor-specific CAF subtype producing ECM components associated with chemoresistance and immune evasion [62]. While the contribution of CAFs to ECM remodeling is increasingly recognized in gastric cancer, further studies are needed to dissect how specific CAF subtypes drive differential collagen deposition and post-translational modifications during distinct stages of tumor progression.

**Table 1:** Upregulation of collagen in gastric cancer. Collagen expressions exhibit atypical expression patterns, as determined by IHC, DNA microarray, RT-PCR, MS, and RNAseq detection methods.

Type	Genes Involved in GC	Normal Gastric Tissue	GC Tissue	Biological Role in GC	References
Collagen I	COL1A1 COL1A2	Moderate (structural support)	High in stromal fibrosis, linked to invasion and metastasis. Potential role as a negative prognostic indicator	Promotes EMT, TME stiffness	[29,41,63,64,65,66,67]
Collagen III	COL3A1	Moderate (maintains mucosal integrity)	Moderate (co-localizes with Collagen I in desmoplasia). Co-overexpression with COL5A2 is proposed as a predictor of poor survival in late-stage GC	Stromal remodeling	[67,68,69,70]
Collagen IV	COL4A1 COL4A2	Moderate (basement membrane)	High. Proposed as a biomarker for poor survival prognosis	Promote EMT, migration, tumor invasiveness, and metastasis	[71,72,73,74]
Collagen V	COL5A1 COL5A2	Moderate	High. Co-overexpression with COL3A1 is proposed as a predictor of poor survival in late-stage GC	Promotes EMT, cell migration	[49,50,69,70,75,76]
Collagen VI	COL6A3	Moderate	Moderate, High. Proposed as a therapeutic target to slow GC progression, blocking specific integrins that bond with Collagen VI	Cell differentiation, migration, and adhesion	[77]
Collagen VII	COL7A1	Moderate	High. Proposed as a biomarker for poor survival prognosis	Tumor invasiveness and metastasis	[78]
Collagen X	COL10A1	Moderate (basement membrane)	High. Proposed as a biomarker for poor survival prognosis and poor immune response. Because of its restricted high expression (only in cartilage and tumor), elevated plasma levels of Collagen X fragments have been found in GC patients	Promotes EMT, tumor vasculature	[48,79,80,81]
Collagen XI	COL11A1	Moderate	High. Proposed as a biomarker for poor survival prognosis and chemotherapy resistance. It has been proposed as a target for CAFs focused therapies in GC	Tumor progression and metastasis, promotes EMT	[62,64,82]
Collagen XII	COL12A1	Non-detected	High. Potential role as a poor survival prognosis. As a therapeutic target, blocking or decreasing Collagen XII can potentially mitigate metastatic	Tumor invasiveness and metastasis	[47]

### 3.2. Collagen Reorganization and Assembly in Gastric Cancer

In most tumor tissues, ECM remodeling is characterized by alterations in the production and deposition of collagen, all accompanied by changes in the expression of remodeling enzymes such as matrix metalloproteinases (MMP), lysyl oxidase (LOX), and lysyl oxidase-like (LOXL) proteins, among others [83].

The tumor ECM is characterized by altered collagen cross-linking, which changes its stability by arranging collagen into fibers with different densities, thereby augmenting ECM stiffness and spatial structure. The deposition of type I collagen fibers is often linked to the survival and spread of tumor cells across different tumor types [37,84,85]. The impact of collagen cross-linking and its relationship with collagen stiffness has been discussed in oral cancer [86].

The increased stiffness in tumors primarily results from extracellular matrix remodeling processes. Factors that regulate ECM remodeling include the activation of CAFs and the accumulation and cross-linking of collagens. Enzymes, including the lysyl oxidase (LOX) family and the lysyl hydroxylase family, are essential in this remodeling process. Lysine hydroxylation and lysine glycosylation are essential for the formation of reducible and mature cross-links generated by the lysyl oxidase system, which dramatically increases tissue tension [33,87,88,89,90]. It has been shown that the elastic modulus of breast cancer tissue can reach 4–12 kPa, dramatically differing from the 0.4–2 kPa of normal mammary tissue [91]. Meanwhile, the matrix experiences major architectural changes. In fact, the porous, randomly oriented collagen meshwork becomes thick and linearized, reducing interstitial pore size from 10–50 μm to 1–10 μm in tumors [92]. This dense structure blocks intratumoral immune effector cell trafficking, such as cytotoxic T lymphocytes, and therapeutic drug perfusion. This condition is a primary cause of chemotherapy and immunotherapy resistance. Microscopic collagen fiber morphology is also altered. Tumor-associated collagen fibers thicken and lengthen, becoming 200–2000 nm in diameter, compared to 50–500 nm in healthy tissue. Importantly, fibers at the tumor’s invasive front are commonly bundled into taut, linear tracks perpendicular to the tumor boundaries. These unique topological properties are defined as tumor-associated collagen signatures (TACS) [46].

In 2017, Zhou et al., validated the prognostic value of collagen in gastric cancer through a systematic analysis of collagen status and content in two independent gastric cancer cohorts in China [29]. The study found that collagen fibers within the stroma of gastric cancer samples were substantially altered, with changes in content, maturity, and fibrillar structure. In particular, collagen fibers showed a marked increase in content, higher linearization, and wider fibrils compared to healthy tissues. They also found a significant increase in immature collagen molecules, together with an increase in CAFs expressing α-SMA and augmented levels of lysyl oxidase-like 2 (LOXL2) in the stroma compared to benign samples, suggesting an increase in type I collagen biosynthesis and crosslinking. This increase could explain the stiffness of the ECM found in many tumors like GC [93,94]. Other studies have found an increase in collagen turnover in gastric cancer tissues, higher levels of collagen types I and IV, fibronectin, and laminin, and higher expression of the COL1α1 gene compared to benign gastric samples, significant enough to discern between malignant and pre-malignant lesions [64,65,95].

In 2021, Liu et al. evaluated the capacity of melatonin to decrease cell proliferation in MGC-803 and SGC-7901 gastric cancer cell lines and found that melatonin directly regulates the production and secretion of matrix metalloproteinase 2 (MMP2) and MMP9 in CAFs, influencing collagen rearrangement in the ECM [96]. Overall, the data suggest that CAFs are crucial contributors to collagen reorganization in gastric cancer, leading to the deposition of fibrillar collagen, and, through the enhanced crosslinking capacity of collagen mediated by stromal LOXL2, promote the stiffness of the gastric tissue around the tumoral gastric cancer cells.

## 4. Collagen Biosynthesis and Degradation in Gastric Cancer

A complex interplay of matrix metalloproteinases (MMPs), tissue inhibitors of metalloproteinases (TIMPs), and lysyl oxidases (LOX) strictly regulates the synthesis, deposition, and degradation of collagen under physiological conditions, thereby maintaining a precise dynamic equilibrium. In contrast, this exquisite equilibrium endures profound dysregulation within the TIME, resulting in the transformation of collagen from a passive structural element to an active driver of malignant progression and therapeutic resistance [97]. Solid tumors often exhibit a desmoplastic reaction, characterized by excessive deposition of fibrillar collagens—especially types I, II, and V, which are upregulated in gastric cancer—aberrant cross-linking and structural remodeling of the extracellular matrix (ECM). This fibrotic response is primarily orchestrated by cancer-associated fibroblasts (CAFs), which are activated through various mechanisms, including transforming growth factor-β (TGF-β) signaling, mechanical stress, and metabolic reprogramming [97,98]. The resulting biomechanical alterations, including the linearization of collagen fibers, tumor-associated collagen signatures (TACS), and increased matrix stiffness, create a pro-tumorigenic niche, facilitating tumor cell invasion and activating mechanotransduction pathways and integrin-FAK signaling that promote proliferation and stemness [99]. The remodeled collagen matrix acts as an immunosuppressive barrier, physically excluding T lymphocytes and immune effectors from the tumor parenchyma. It also delivers inhibitory signals, suppressing T cell activation and promoting the polarization of tumor-associated macrophages (TAM) toward an M2-like phenotype [99]. Through the signal transducer and activator of transcription 3 (STAT3) and nuclear factor kappa-B (NF-κB) pathways, inflammatory cytokines and reactive oxygen species produced during frustrated immune responses paradoxically increase CAF activation and collagen production, resulting in a self-reinforcing cycle [100].

**Figure 2:**
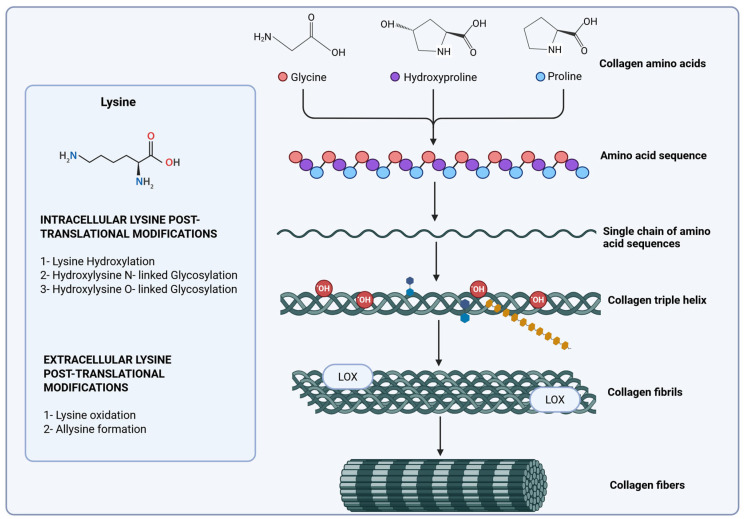
Key post-translational modifications (PTMs) in collagen biosynthesis. This schematic illustrates major PTMs critical for collagen structure and function, that occur primarily within the endoplasmic reticulum (ER) and extracellular space. The figure shows the key chemical changes collagen proteins undergo after they are made. First, inside the cell, specific proline and lysine building blocks in the collagen chain are hydroxylated (adding -OH groups) by enzymes. Hydroxylated proline (4-hydroxyproline) is crucial for the collagen’s stable triple-helix shape. Hydroxylated lysine (hydroxylysine) then allows glycosylation—the attachment of sugar molecules (like galactose or glucose-galactose)—which helps control the size and spacing of collagen fibers. After the collagen is secreted and forms fibers outside the cell, the enzyme lysyl oxidase (LOX) oxidizes specific lysine and hydroxylysine residues, mainly at the ends of the molecules. This oxidation creates reactive groups that form strong covalent cross-links between collagen molecules, giving the final tissue its essential strength and stability. The figure was created with the license of BioRender.

Post-translational modifications play a crucial role in the stabilization of collagen, involving complex processes such as the hydroxylation of proline, which is vital for maintaining the integrity of the triple-helical structure. Additionally, lysine hydroxylation serves as a prerequisite for the subsequent O-glycosylation of hydroxylysine, a key modification necessary for proper extracellular matrix deposition and assembly. This process is facilitated by the formation of physiological collagen cross-linking, which is catalyzed by lysyl oxidase enzymes. These enzymes perform oxidative deamination of lysine and hydroxylysine residues in the telopeptide regions, leading to the establishment of both intra- and intermolecular covalent cross-linking (Figure 2) [85,101,102]. Enzymes that break down proteins, such as matrix metalloproteinases (MMPs), and enzymes that support collagen fiber cross-linking, such as lysyl oxidases (LOX and LOXL2), work together to change the ECM composition and quality through modifications in fiber assembly [42,103,104]. Nonetheless, although recent research has highlighted the importance of collagen fiber formation in the advancement and metastasis of gastric cancer, the alterations in cross-linking and assembly, as well as the activity of collagen-biosynthetic enzymes during the progression of metastatic gastric cancer, remain inadequately comprehended [3,29,39,40].

A central mechanism in gastric cancer stromal remodeling is the hypoxia-induced activation of collagen-modifying enzymes, as observed in gastric cancer and other tumor types. Activation of HIF-1α in the hypoxic microenvironment upregulates the expression of lysyl oxidase (LOX and LOXL2) and lysyl hydroxylases such as PLOD2 [105,106,107], leading to abnormal maturation of type I and III collagen fibers through cross-linking and covalent stabilization. The expression of matrix metalloproteinases (MMPs)—particularly MMP2 and MMP9—is significantly increased in advanced-stage tumors [108,109,110], enabling focal ECM degradation, basement membrane disruption, and enhanced cellular migration, contributing to epithelial-mesenchymal transition [111]. Importantly, experimental inhibition of LOX (e.g., via BAPN) significantly reduces both the expression and enzymatic activity of MMP-2 and MMP-9 [112], whereas in non-small-cell lung and triple-negative breast carcinoma, exogenous LOX elevates MMP levels in a dose-dependent manner progression [112,113,114], indicating a sequential regulatory axis between LOX upregulation and MMP activation in gastric cancer. In this context, the functional synergy between LOX, PLOD2, and MMPs creates a microenvironment favorable for tumor invasion, where stiffness precedes localized enzymatic degradation of the extracellular matrix, facilitating cell migration and intravasation. This hypoxia–matrix remodeling enzyme–ECM architecture axis represents a valuable conceptual framework for understanding stromal contributions to gastric cancer progression and may offer a relevant therapeutic target. The following subsections detail the intracellular and extracellular collagen biosynthetic enzymes involved in the crosslinking, assembly, and degradation of collagen fibers, as well as the anomalies in collagen crosslinking caused by advanced glycation end products (AGE products).

### 4.1. Collagen Hydroxylases

Proline residues within the Y position of Gly-X-Y triplets are hydroxylated by prolyl 4-hydroxylase (P4H) to form 4-hydroxyproline (Hyp). This modification is essential for triple helix stability at physiological temperatures. Specific lysine residues within the Y position are hydroxylated by lysyl hydroxylases 1, 2, and 3 (LH 1–3), encoded by PLOD 1–3 genes, respectively. Of these enzymes, LH1 and LH3 hydroxylate to lysine residues located in the helical domain, while LH2 hydroxylates lysine residues located in the collagen telopeptide domains to form 5-hydroxylysine (Hyl). This is a prerequisite for glycosylation and influences cross-linking.

Hyl can be glycosylated within the ER by specific glycosyltransferases. Galactosylation is the addition of a galactose (Gal) moiety that forms galactosyl-hydroxylysine (Gal-Hyl). Glucosyl-galactosylation is the addition of glucose (Glc) to Gal-Hyl, forming glucosyl-galactosyl-hydroxylysine (Glc-Gal-Hyl). Glycosylation modulates fibril diameter and organization (Figure 2).

In cancer tissues, cross-linking is driven toward the hydroxylysine aldehyde pathway because of the overexpression of LH2 enzyme, promoting ECM stiffness. While this pathway has not been explored in gastric cancer genesis or progression, several reports suggest LH2 overexpression as a potential biomarker and therapeutic target [115,116]. The PLOD2 gene has been found to be upregulated in cancer, particularly in response to hypoxia and TGF-β1, and is associated with cancer progression and metastasis [117,118,119]. PLOD2 expression has also been shown to be a prognostic indicator in various cancers, including gastric cancer and osteosarcoma [95,120]. Furthermore, PLOD2 has been found to play a role in immune cell infiltration in osteosarcoma, suggesting its potential as a target for immunotherapy. PLOD2 regulated by HIF-1 is a potential regulator of peritoneal dissemination of gastric cancer [121]. The authors showed that PLOD2 promotes cell invasiveness and migration in gastric cancer under hypoxia and leads to peritoneal dissemination of gastric cancer [121].

### 4.2. Lysyl Oxidases

Following secretion and fibril assembly, specific lysine and hydroxylysine residues in the telopeptide regions (N- and C-terminal nonhelical ends) are oxidatively deaminated by lysyl oxidase (LOX) enzymes. This reaction generates reactive aldehydes (allysine and hydroxyallysine). These aldehyde groups spontaneously react with neighboring unmodified lysine/hydroxylysine residues or other aldehydes to form covalent intra- and intermolecular cross-links, providing the fibrillar network with critical tensile strength and mechanical stability in the ECM [122].

This mechanism enables the creation of intramolecular cross-links, which are essential for the proper development of skin, bones, the aorta, and other tissues. Regrettably, the antagonistic pleiotropy theory of aging posits that the enzymatic activity crucial in youth may become suboptimal throughout the course of life. Excessive lysyl oxidase contributes to increased stiffness in solid tumors and enhances the survival of metastatic cells. Lysyl oxidase (LOX) catalyzes collagen crosslinking in the tumor-associated ECM. Studies show that the family of enzymes called LOX is key in the crosslinking of collagen molecules and, therefore, plays an important role in the ECM remodeling process [123].

Several studies have reported changes in LOX, LOXL1, LOXL2, LOXL3, and LOXL4 expression in gastric cancer [104,124,125,126]. Evidence related to the LOX protein shows that its expression is significantly associated with invasion depth, tumor differentiation, lymph node metastasis, lymphatic invasion, venous invasion, and peritoneal metastasis [111]. Studies have reported that the LOX protein is highly expressed in gastric cancer tissues, and its expression has been correlated with poor overall survival [126]. The overexpression of this protein has been associated with invasion, migration, and EMT processes that occur in gastric cancer [111]. A study shows that LOX overexpression is significantly correlated with T-stage progression in gastric cancer. High expression of LOX is related to ECM receptor interaction, cancer, Hedgehog, TGF-beta, JAK-STAT, MAPK, Wnt, and mTOR signaling pathways [126]. Studies show that LOX, MMP-2, and MMP-9 act on the ECM by remodeling it. Studies indicate that the expression of both MMP-9 and MMP-2 correlates with the expression of LOX in gastric cancer biopsies [112,127].

Regarding LOXL1 in gastric cancer, one study indicates that LOXL1 has been associated with peritoneal dissemination, which frequently occurs in gastric cancer. Evidence described in this study suggests that this process would be driven by the induction of EMT [128]. The expression of LOXL1 was correlated with T invasion, lymph node metastasis, and lymphatic and venous invasion in gastric cancer samples [124]. Interestingly, LOXL1 expression has been associated with gastric cancer with intestinal-type histology. Kasashima et al., 2018, reported that TGF-β1 decreased the expression of LOXL1 in diffuse-type gastric cancer cell lines [124].

In gastric cancer, peritoneal dissemination (PD) frequently occurs, causing a poor survival prognosis. Qingjiang Hu et al. studied the relationship between LOXL1 and PD in gastric cancer using immunochemical techniques and gene expression analysis [128]. It was shown that the overexpression of LOXL1 can induce epithelial–mesenchymal transition (ETM) in gastric cancer cells, thus causing cell migration and metastasis, and is associated with tumor cells with a poorly differentiated histological type and with PD in gastric cancer, suggesting LOXL1 as a biomarker and a potential therapeutic target in gastric cancer.

Studies show that the abnormal expression of LOXL2 plays an important role in the tumor progression of several types of cancer [125,129]. A study in gastric cancer biopsies shows that the expression of the TRIM44 protein is inversely correlated with the expression of LOXL2. This study shows that TRIM44 regulates the stability of LOXL2 and thus modulates the ability of LOXL2 to remodel the extracellular matrix in gastric cancer [130].

Regarding LOXL3, it is known that the expression of this protein is increased in pancreatic ductal adenocarcinoma cells and melanoma, among others [131,132]. In gastric cancer, the information is not abundant. A study indicates that the expression of LOXL3, like other members of this protein family, was correlated with tumor invasion, lymph node metastasis, and poorer prognosis of patients. Additionally, this study describes TGF-induced LOXL3 upregulation in gastric cancer cells, suggesting that LOXL3 is downstream from the TGF-signaling pathway [124].

In relation to LOXL4, changes in its expression have also been described in various types of cancer. An increase in its expression has been correlated with metastasis to lymph nodes and high-grade tumors [133,134]. In gastric cancer, it has been described that LOXL4 expression is upregulated in biopsies. This increase in expression has been correlated with tumor size, depth of tumor invasion, lymph node metastasis, and poorer survival [135]. Studies in gastric cancer cell lines show that LOXL4 promotes cell proliferation, migration, and invasion through the FAK/Src pathway [135,136].

### 4.3. Matrix Metalloproteinases (MMPs)

**Figure 3:**
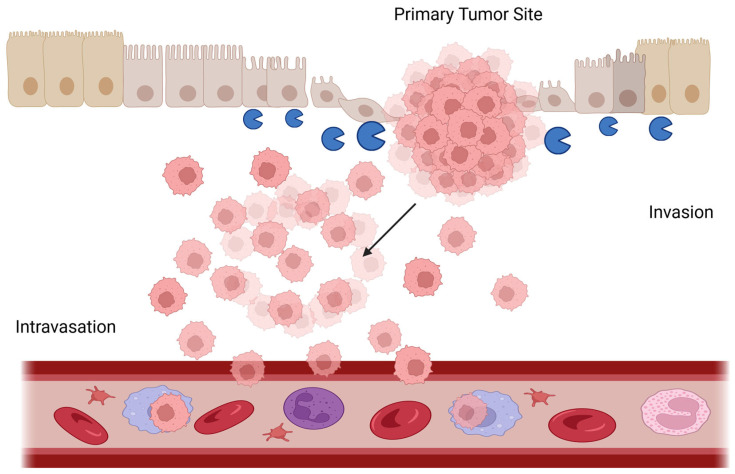
Metastatic invasion and intravasation: This schematic representation illustrates key steps in cancer metastasis. ECM degradation by MMPs: Tumor cells (orange) secrete matrix metalloproteinase (MMPs) enzymes that degrade the extracellular matrix (ECM) and basement membrane. This enzymatic breakdown enables tumor cells to detach and invade surrounding tissues. Intravasation: Motile tumor cells migrate through the disrupted ECM toward a blood vessel (red). They then undergo intravasation—entering the bloodstream (red blood cells)—via endothelial cell junctions. The figure was created with the license of BioRender.

While an increase in collagen cross-linking and ECM stiffness promotes tumoral growth and progression, the degradation of the collagen fibers is a key step when tumoral cells begin the advanced or metastatic stage of many cancers, including gastric cancer [137,138]. Matrix turnover is organized by several enzymes, with MMPs being the major class of matrix-degrading proteinases responsible for that role [139]. Human MMPs constitute a family of 23 calcium-dependent, zinc-containing endopeptidases that play a pivotal role in regulating EMC homeostasis, releasing bound growth factors like VEGF, and activating intracellular signaling responses [140]. MMPs restructure the ECM and facilitate tumor advancement and metastasis through many mechanisms, including the disruption of the basement membrane, downregulation of E-cadherin, and the stimulation of EMT (Figure 3) [140].

In gastric cancer, the roles of MMP2 and MMP9 have been described extensively in the literature compared to other MMPs, with both enzymes proposed as novel biomarkers for gastric cancer prognosis, indicating a major role in the gastric cancer metastatic process [141]. Studies about the impact of *Helicobacter pylori* infection on gastric cancer cells show an increase in MMP2 and MMP9 activity, inducing ECM remodeling and cell invasion, while a comparative study of both enzymes shows a significant increase in expression in intestinal-type gastric cancer compared to diffuse type gastric cancer [142].

Several meta-analyses and bioinformatic studies have found that the overexpression of MMPs, like MMP2 and MMP9, is associated with a poor prognosis in gastric cancer patients. MMP9 has been positively correlated with the depth of gastric cancer invasion, and higher levels of MMP9 have been reported in the serum of gastric cancer patients compared to controls. In other tumors, exosomes rich in matrix-degrading enzymes can facilitate cancer cell dissemination to both primary and metastatic sites, suggesting that the MMP9 serum of gastric cancer patients could be related to the metastatic progression of the disease.

It is well established that MMP-2, MMP-9, and MMP-11 contribute to the remodeling of the tumor microenvironment (TME) in gastric cancer through both structural and functional mechanisms. MMP-2 and MMP-9 degrade key ECM components—such as collagen IV, laminin, and fibronectin—facilitating basement membrane disruption, increased matrix porosity, and enhanced tumor invasion [143]. However, beyond matrix degradation, MMPs also exhibit a profibrotic role by liberating ECM-bound factors like TGF-β, which induce the activation of cancer-associated fibroblasts (CAFs) and promote the synthesis of fibrillar collagens, contributing to desmoplastic matrix stiffening [144].

MMP-11, primarily expressed by CAFs, targets non-ECM substrates such as α1-antitrypsin, indirectly promoting ECM remodeling and tumor cell migration [59,144]. Moreover, MMPs support angiogenesis through the release of VEGF and modulate immune infiltration by altering chemokine gradients and generating bioactive ECM fragments, as has been thoroughly described by Quintero-Fabián et al., 2019 [145]. These dual actions—initial matrix breakdown followed by fibrotic remodeling—illustrate how MMPs orchestrate both architectural and signaling changes in the TME that favor gastric cancer progression.

### 4.4. Collagen Cross-Linking by Advanced Glycation Products

Advanced glycation end products (AGEs) are proteins or lipids glycosylated non-enzymatically by glucose or other reducing sugars [146]. It has been widely reported that AGEs disrupt normal protein function and can cause protein aggregation, a phenomenon that has been shown to promote the transformation of healthy cells into tumor cells [147]. For example, studies suggest that AGEs increase muscle rigidity, promoting cross-linking between collagen and elastin, which alters muscle function [148]. Along the same lines, studies show that in skin exposed to the sun, the normal fibrillar pattern of the extracellular matrix is replaced by trivalent collagen crosslinks, causing thickening and disorganization of the collagen fibers [149].

Among the different components of the extracellular matrix, type I collagen is the most abundant protein [150]. Due to the longevity of this protein in tissues (15 years in humans) collagen I is a prominent target of non-enzymatic post-translational in vivo modifications such as carbamylation and glycation [151]. A study in HT1080 cells shows that collagen I glycation can affect the migration of tumor cells in vitro [151]. It has been described in keratinocytes and lung cells that AGE-modification of collagens I and IV affects their interaction with other components of the matrix, altering cell migration and adhesion [152,153]. Glycation, with cell adhesion to type I collagen, and collagen cross-linking increase during aging and in tumors as a product of non-enzymatic glycation, affecting its ability to bind to other elements of the extracellular matrix [154]. This study shows that the glycation of collagen fibers produces significant changes in the charge and molecular arrangement of the fibers studied, affecting the interaction of extracellular matrix elements [154,155]. A very recent study investigated the impact of AGEs on the stability and functionality of type I collagen, revealing the biochemical evidence that glycation, induced by ribose, leads to decreased triple helical stability, increased molecular stiffness, and impaired self-assembly of collagen fibrils, thereby elucidating the molecular mechanisms by which glycation contributes to age-related tissue alterations [156]. A study on malignant breast tumor cells showed that glycation of collagen matrices favors tumor cell invasion and migration, particularly in spheroids [157].

Evidence shows that protein glycation is one of the molecular events that accompany many oncogenic transformations, including in gastric cancer [158]. Although evidence of collagen glycation in gastric cancer models is scarce, a study evaluated the expression of AGE receptors (RAGE) in various gastric carcinoma cell lines and their association with invasion and metastasis [159]. The study describes that almost all cell lines constitutively express RAGE. Through immunohistochemistry, the study found that a high percentage of the gastric cancer biopsies studied were positive for RAGE. The study shows that RAGE-positive cancer cells tend to be distributed at the invasive front of the primary tumors and are detected in all metastatic lymph node foci. RAGE expression appears to be closely associated with invasion and metastasis in gastric cancer [159]. Binding of AGEs to RAGE on immune and stromal cells (e.g., macrophages, cancer-associated fibroblasts) activates pro-inflammatory pathways (notably NF-κB). This drives the sustained production of cytokines (e.g., TNF-α, IL-6), chemokines, and growth factors, fostering a chronic inflammatory tumor microenvironment. This inflammation promotes cancer cell proliferation, survival, angiogenesis, and immune evasion.

**Figure 4:**
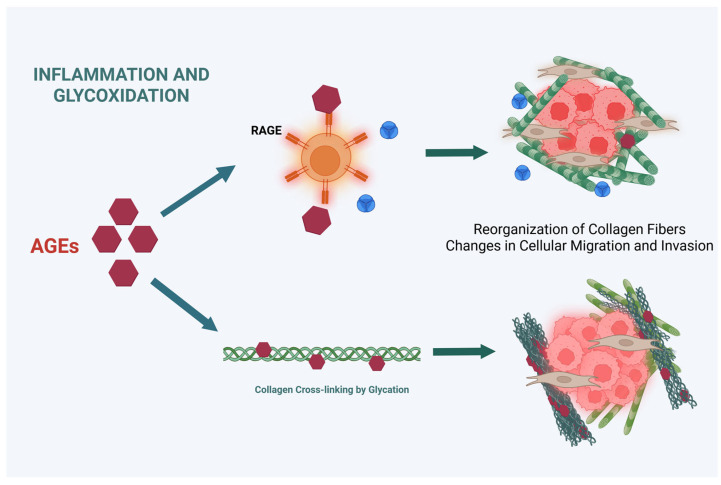
Dual impact of advanced glycation end products (AGEs) in gastric cancer: RAGE-driven inflammation and collagen glycation in the tumor microenvironment. This figure illustrates how advanced glycation end products (AGEs) drive gastric cancer progression through two interconnected mechanisms: (1) Binding of AGEs to their receptor (RAGE) activates pro-inflammatory signaling (e.g., NF-κB) in tumor and stromal cells and sustains a cytokine-rich environment that promotes cancer growth and invasion. (2) Non-enzymatic glycation directly modifies collagen in the tumor’s extracellular matrix (ECM), forming stiff, cross-linked fibers that impair tissue remodeling and facilitate metastasis. Critically, glycated collagen itself becomes a source of new AGEs, creating a damaging feedback loop: AGE-RAGE inflammation accelerates collagen glycation, and glycated collagen fuels further RAGE-driven inflammation, collectively worsening cancer progression [162]. The figure was created with the license of BioRender.

Previous research has shown that collagen glycosylation is involved in the processes of cell migration and metastasis in various types of cancer. In relation to gastric cancer, alterations in cadherins and mucins have been described [160,161]. To date, there are no studies that have investigated the potential effects of collagen glycation on the extracellular matrix in gastric cancer. However, non-enzymatic glycation modifies collagen fibers within the tumor-associated ECM. AGEs form irreversible cross-links between collagen molecules, leading to ECM stiffening, impaired remodeling, and altered signaling, and the glycated collagen itself could act as a source of AGEs, further fueling RAGE activation and inflammation [162]. This could create a detrimental feedback loop between AGE-modified ECM and chronic inflammation, driving gastric cancer progression and metastasis (Figure 4).

## 5. Decellularized Tissues as Scaffolds for Biomimetic 3D In Vitro Models in Gastric Cancer

In recent years, three-dimensional (3D) models have gained prominence in the field of regenerative medicine and cancer research, especially through the use of decellularized extracellular matrices (dECM) and organoids. Pennarossa et al. (2022) [163] highlighted that organ decellularization allows for the obtaining of natural biological scaffolds that preserve the architecture and biomechanical properties of the original tissue, despite the technical challenges associated with the complete removal of genetic material and the functional preservation of the matrix. These advances are relevant, as traditional 2D cell culture systems have critical limitations in that they do not adequately reproduce the in vivo cellular microenvironment, which affects the validity of preclinical results and contributes to the high failure rate in the development of new drugs [163].

In the oncology context, Neal et al. (2018) [164] proposed a model based on patient-derived organoids that integrate both tumor cells and autologous immune elements. This system allows for the study of the interaction between tumor-infiltrating T cells (TILs) and tumor cells under physiologically relevant conditions, representing a significant advancement in the evaluation of immunotherapies such as PD-1/PD-L1 blockade. The fidelity of the model was supported by single cell sequencing analyses, which confirmed the conservation of dominant T cell clones and the expression of genes associated with cellular exhaustion, opening opportunities for personalized approaches in immunotherapy.

Similarly, Varinelli et al. (2023) [165] developed a 3D dECM model derived from neoplastic peritoneum to study peritoneal metastases in colorectal cancer. This system faithfully reproduces the biomechanical and molecular conditions of the metastatic niche, including increased rigidity, a high concentration of glycosaminoglycans, and the expression of genes associated with drug resistance. Furthermore, the tumor organoids cultured in these matrices showed increased resistance to conventional treatments such as oxaliplatin, highlighting the active role of the tumor microenvironment in therapeutic response. Although limited by the absence of immune and vascular components, the model represents a valuable tool for pre-clinical studies and the design of personalized therapies.

On the other hand, the immunomodulatory potential of decellularized matrices has also been explored in non-tumoral tissues. Gvaramia et al. (2022) [166] demonstrated that decellularized porcine cartilage (DNSC) can induce mixed responses in human macrophages, combining both M1 inflammatory and M2 anti-inflammatory markers. However, by functionalizing the material with IL-4, it was possible to induce a dominant anti-inflammatory profile and restore the migration of progenitor cells, significantly improving the biocompatibility of the biomaterial. This strategy offers a promising avenue for optimizing cartilage implants in regenerative therapies.

Complementarily, Deng et al. (2023) [167] designed a hydrogel functionalized with extracellular vesicles derived from mesenchymal stem cells (MSCs) on a decellularized spinal cord matrix. This combination allowed the reprogramming of macrophages toward an M2 phenotype, reducing inflammation and promoting neuronal regeneration in vitro. Although the results were obtained in murine models, the authors suggest that this strategy could be applied to other inflammatory neurological diseases, such as multiple sclerosis, opening new possibilities in advanced therapies with an immunological and regenerative focus.

Taken together, these studies underscore the strategic value of decellularized matrices and 3D models as versatile platforms for studying the tissue microenvironment in both regenerative and tumoral contexts. The possibilities they offer in terms of personalization, physiological recapitulation, and immune response analysis consolidate their relevance as key tools in the development of advanced translational medicine.

### 5.1. Comparison of Features of dECM Sources

Advanced in vitro preclinical models are necessary to better understand tumor progression and therapeutic responses because gastric cancer continues to be one of the top causes of cancer-related mortality globally [1]. Gastric cancer has emerged as one of the most lethal malignancies, often diagnosed at an advanced stage, thereby limiting the efficacy of therapeutic interventions in the primary site of the tumor to avoid metastasis. ECM plays a crucial role in tumor initiation and progression by influencing cell adhesion, survival, proliferation, migration, and signaling pathways [168]. To understand the roles of ECM in gastric cancer, researchers have focused on single or multiple ECM molecules and have examined the effects of these on cell behavior using genetically mutated cells/animals and substrates coated with ECM like Matrigel. However, ECM is the assembly of many molecules—collagens, for example—and this protein activates multiple signaling pathways which, when orchestrated inside the cells, exhibit specific cell behaviors. While animal models frequently lack pathological relevance, particularly to humans, traditional two-dimensional (2D) cell cultures are unable to replicate the complex TME. In this context, decellularized extracellular matrix (dECM)-based three-dimensional (3D) biomimetic in vitro models have become a viable substitute to close this gap [169]. These platforms could offer a more physiologically appropriate system for researching the biology of gastric cancer, drug screening, and customized treatment by maintaining natural tissue architecture and biochemical signals.

There are two primary sources for obtaining dECM; one is derived from native tissues and organs of the body, and the other is sourced from regenerated tissues and organs constructed from cultured cells [19,20,21,22,23]. Consequently, dECM is typically classified into two categories: tissue/organ-derived dECM and cultured cell-derived dECM. Each approach presents various advantages and disadvantages [170]. Tissue/organ-derived dECM should have a similar composition and microstructure to native ECM. However, culture mediums, initial substrates, culture periods, cell types, and passage numbers can easily change the content and architecture of cultured cell-derived dECM [170]. The biggest benefit of growing cell-derived dECM is its similarity to natural ECM. If properly prepared, cultured cell-derived dECM can recreate natural ECM in a stem cell niche [42,43].

Reconstructing ECM in limited locations is problematic for tissue/organ-derived dECM because these regions are hard to identify and isolate. Tissues and organs for dECM preparation are scarcer than cultured cells. Low sample numbers make large-scale cell and molecular biology analysis of intracellular signaling in tissue/organ-derived dECM problematic. Tissue/organ-derived dECM is difficult to use for cancer research since malignant ECM composition and microstructure vary by patient. Native ECM composition and microstructure vary even among cancer patients. This causes significant batch-to-batch variation in tissue/organ-derived dECM. Carefully choosing dECM sources for cancer research is necessary.

Although both dECM and Matrigel are utilized as three-dimensional scaffolds in tissue engineering and cell culture, their origins, compositions, and characteristics are very different (Table 2). Whereas Matrigel is a commercially available basement membrane extract from a mouse sarcoma, dECM is produced from native tissues, maintaining tissue-specific properties.

### 5.2. Techniques for Decellularization for Obtaining dECM Materials

Decellularization techniques remove cellular components while retaining structural and functional ECM proteins (e.g., collagen, fibronectin, glycosaminoglycans), creating scaffolds that are able to mimic the native gastric TME (dECM) [171]. The decellularization process for tissues and organs is very carefully designed to remove as many cellular components as possible while keeping the native ECM’s most important parts, biological activity, and structural integrity [172]. Decellularization is an essential step in the preparation of dECM, as it greatly influences its microstructure and composition. There are several methods that are used frequently to eliminate cellular components, namely physical, chemical, and enzymatic techniques [173]. These strategies have their own advantages and disadvantages when compared to each other. As a result, they are usually used together to address their limitations and variability. For example, chemical strategies like detergents such as sodium dodecyl sulfate (SDS), sodium deoxycholate, and Triton X-100 may be considered [170]. These detergents solubilize cytoplasmic and nuclear lipid membranes and proteins, enabling effective decellularization; however, they often compromise the ECM microstructure, resulting in a partial loss of ECM components. Alkaline and acid solutions are also chemical decellularization methods, similar to detergents, as they solubilize nucleic acids and components of the cytoplasm; however, they do reduce glycosaminoglycans. On the other hand, some severe chemicals, including sodium sulfide and sodium hydroxide, can break up collagen crosslinks, which makes dECM much weaker [174]. In addition, hypotonic and hypertonic solutions induce cell lysis and dehydration through osmotic effects; however, it is challenging to eliminate DNA remnants [172].

Second, physical methods are employed, such as freeze–thaw cycles, which can disrupt cell membranes while also fracturing the ECM microstructure because they induce the formation of intracellular ice crystals [175]. Mechanical pressure facilitates decellularization by assisting in the separation of cells from the underlying basement membrane, and ultrasound strategies disrupt the cell membrane structures [176]. Supercritical fluid technology represents an innovative method for cell removal that is environmentally friendly, utilizing carbon dioxide as a green solvent, thereby eliminating the necessity for hazardous chemicals. In a supercritical carbon dioxide environment, the lipid components of cell membranes undergo significant degradation, facilitating effective decellularization [172]. Previous research indicated that dECM materials produced through supercritical fluid technology retained ECM components more effectively than conventional chemical methods [177]. However, for complete purification, additional washing and enzymatic treatment processes may be required, as physical treatment alone might not completely remove the remaining cell membranes and cellular remnants. The enzymatic treatment presents a possible method for dECM preparation by disrupting cell-to-cell and cell-to-ECM interactions through the application of nucleases, proteases, collagenases, lipases, and other enzymes [172,173]. This process facilitates the targeted removal of cellular remnants and effective cell eradication.

Finally, the solubilization of dECM with pepsin is utilized to form a gel, which is an effective method for obtaining homogeneous samples; however, it is important to note that the microstructure may be compromised in this case [178]. Notably, enzymatic methods often result in considerable damage to extracellular matrix components, which diminish the mechanical strength of the ECM.

### 5.3. Studies in Cancer Initiation and Progression Using dECM

Pre-clinical gastric cancer phenotype, aggressiveness, metastasis, and pharmaceutical discovery studies generally use 2D in vitro cancer cell culture models. Matrigel has been extensively researched to unveil the role of ECM molecules in cancer initiation and progression. However, there are few studies that thoroughly investigate the roles of assembled collagen molecules in cancer progression. Additionally, there are very few studies that concentrate on collagen remodeling [170]. To comprehend the roles of ECM in cancer initiation and progression, one must consider the compositional and structural dynamics of the ECM through the lens of assembled ECM molecules. In vitro ECM models will be beneficial for this purpose.

Liu and colleagues decellularized human breast cancer biopsy tissues using SDS, recellularized them with MCF-7 cells, and found that it enhanced cell proliferation, migration, EMT, and resistance to 5-fluorouracil [179]. Zhao and colleagues used transfection techniques to alter lysyl oxidase expression in MDA-MB-231 cells, resulting in tumors with varying ECM stiffness when implanted in animals. After decellularization and recellularization, scaffolds with higher stiffness showed increased cisplatin resistance and drug-resistant gene expression [180]. Researchers have created patient-derived scaffolds from breast cancer specimens and recellularized them with various cell lines, including MCF-7, T-47D, and MDA-MB-231, to offer different in vivo-like microenvironments for drug screening [181]. The scaffolds showed heightened resistance to drugs like 5-fluorouracil, doxorubicin, and paclitaxel, while T-47D and MDA-MB-231 cells exhibited growth patterns similar to MCF-7 cells. The scaffold’s microenvironment influences drug responses, with different cell lines affecting specific gene expression in chemotherapy.

In order to produce in vitro three-dimensional scaffolds for cell growth, Agostini et al. recently decellularized pancreatic cancer tissues taken from several patients via surgical excision at a hospital [182]. Mass spectrometry analysis showed chemical differences compared to normal pancreatic tissue, indicating that this scaffold accurately replicated the ultrastructure of the pancreatic tumor microenvironment. Interestingly, this 3D tumor model confirmed the increased cytotoxicity of Folfirinox regimens for pancreatic cancer and demonstrated resistance to 5-fluorouracil compared to 2D cells.

Cultured cell-derived dECM is employed in cancer research. From the mouse normal fibroblast NIH-3T3 cell line and cancer-associated murine fibroblasts, Cukierman and colleagues generated 3D dECM. They chose fibroblasts as a tissue ECM source because they produce many ECM compounds. Cell phenotypes and intracellular signaling were evaluated in 3D dECM generated by normal and cancer-associated fibroblasts. In 3D dECM, they cultured breast cancer MCF-7, MDA-MB-231, and MCF-10A cells. In particular, Akt activation increased spindle morphologies in cancer-associated fibroblast-derived dECM in MDA-MB-231 cells [170].

Various types of dECM have been proposed to investigate cell behaviors and elucidate the roles of ECM in cancer. dECMs were prepared from both normal and cancerous tissues, revealing distinct cellular behaviors attributed to compositional and structural differences between the two types of dECM [170]. Cancerous dECM appears to enhance cell migration, angiogenesis, and EMT responses. Therefore, utilizing cancerous dECM as a model for native cancerous ECM may be appropriate for examining the functions of ECM in cancer at primary sites.

### 5.4. dECM Scaffolds to Study Collagen Assembly in the Gastric Tumor Microenvironment

Decellularized matrices offer a primary benefit in their structural and biochemical fidelity to native tissues, crucially retaining region-specific ECM features like collagen architecture [183]. This preservation is particularly significant for modeling gastric cancer, where studies demonstrate that decellularized scaffolds present the characteristic collagen reorganization—increased linearization and alignment—observed in the TME, which facilitates cancer cell invasion. Researchers employ these matrices to develop cancer models that are pathologically relevant by repopulating them with various cell lines, including those from gastric cancer. This technique demonstrates significant benefits, allowing for the examination of cancer progression and treatment responses ex vivo without depending exclusively on live subjects. The process further allows the creation of customizable models tailored to specific research needs, such as investigating stage-specific TME dynamics. As a result, decellularized tissues have become a valuable resource in cancer research, allowing for a close look at how cells interact with the ECM in the TME, especially how collagen works, and offering a realistic setting to effectively evaluate how well treatments work.

Gastric cancer models that accurately mimic tumor-specific cellular and matrix microenvironments are being created using innovative tissue engineering methods. Tissue-engineering methods grow cancer cells in synthetic porous polymer scaffolds or natural collagen/Matrigel hydrogels to build tumor tissue. These models can overcome 2D monolayer culture restrictions by simulating cancer growth, progression, and metastasis using more similar matrix conditions. However, synthetic polymer scaffolds like PLA and PLGA, as well as natural collagen/Matrigel, fail to duplicate the stomach’s extracellular matrix (ECM). On the other hand, decellularized rat stomachs have been studied for human medicine. Rat stomachs lack submucosal glands in the esophagus, unlike pigs and humans. Thus, replicating the human stomach using the rat model would miss important anatomical traits.

However, dECM from swine or human stomach tissue provides species- or patient-specific matrices for biomimicry. Unlike synthetic hydrogels with Matrigel, dECM creates a tumor-relevant mechanical and biochemical milieu that promotes cancer cell proliferation, invasion, and treatment resistance almost in vivo [184]. The 3D dECM-based models enable the study of tumor–stroma interactions, EMT, and metastasis. Co-culture systems incorporating CAFs, immune cells, and endothelial cells within dECM scaffolds replicate the heterocellular crosstalk observed in tumors [185].

Porcine extracellular matrix (ECM) is often considered an excellent model for studying collagen due to several key advantages, such as structural and biochemical similarity to human collagen. In fact, porcine (pig) collagen has a high degree of homology with human collagen in terms of amino acid sequence, fiber organization, and cross-linking patterns. In addition, the triple-helical structure of porcine type I collagen closely resembles that of humans, making it highly relevant for biomedical research. Porcine skin and tendons are rich in type I collagen, the predominant collagen in human skin, bones, tendons, and scars. This makes porcine ECM a representative model for studying wound healing, tissue engineering, and fibrosis. Also, its mechanical properties mimic human tissues. It has been described that porcine ECM has similar tensile strength, elasticity, and degradation kinetics compared to human collagen, making it ideal for biomechanical studies (e.g., tendon/ligament research) and scaffold development for tissue engineering. Porcine-derived collagen is already used in wound dressings due to its biocompatibility and in the decellularized ECM scaffolds for regenerative medicine. This means research findings can be directly translated into clinical applications. Additionally, it presents ethical and practical advantages over human or other animal models because it is more readily available than human cadaver-derived collagen. It has a lower risk of disease transmission compared to bovine (cow) collagen (e.g., no risk of prion diseases like BSE) and better standardization—porcine tissues are more uniform than human donor tissues, reducing experimental variability.

Kim et al. created a new bioink that combines gastric decellularized extracellular matrix (dECM) and cellulose nanoparticles (CN) to make a stronger material that can mimic the chemical environment found in gastric cancer [186]. Research indicates that collagen nanofibers (CN) might enhance the mechanical properties of the matrix, including stiffness, which may facilitate the advancement of gastric cancer, as it is hypothesized that the behavior of cancer cells is influenced by extracellular matrix (ECM) stiffness. It was confirmed that gastric cancer cells developed larger aggregates as a result of increased stiffness of the extracellular matrix (ECM) [186].

Additionally, a modified decellularization protocol effectively removes cellular material yet preserves the porcine esophageal matrix’s microstructure, making it suitable for cell growth and migration studies in esophageal cancer models [184]. In addition, three-dimensional biomimetic cell culture platforms utilizing COL4 pre-treated hydrogels have been found to support the proliferation of glioblastoma cells [187]. Shigeta et al. reported an optimized method for obtaining decellularized gastric tissue from porcine sources. The researchers systematically analyzed different anatomical regions of the stomach—including the fundus, body, and antrum—to evaluate the preservation of region-specific ECM components, including collagen and elastin content [188]. The decellularized tissue underwent comprehensive biochemical characterization (confirming >95% DNA removal and partial glycosaminoglycan loss while retaining collagen/elastin) and mechanical testing (revealing increased elastic modulus but reduced tensile strength). Notably, the researchers repopulated the scaffolds with HepG2 cells, demonstrating cytocompatibility through sustained cell proliferation of the acellular porcine gastric tissue.

**Figure 5:**
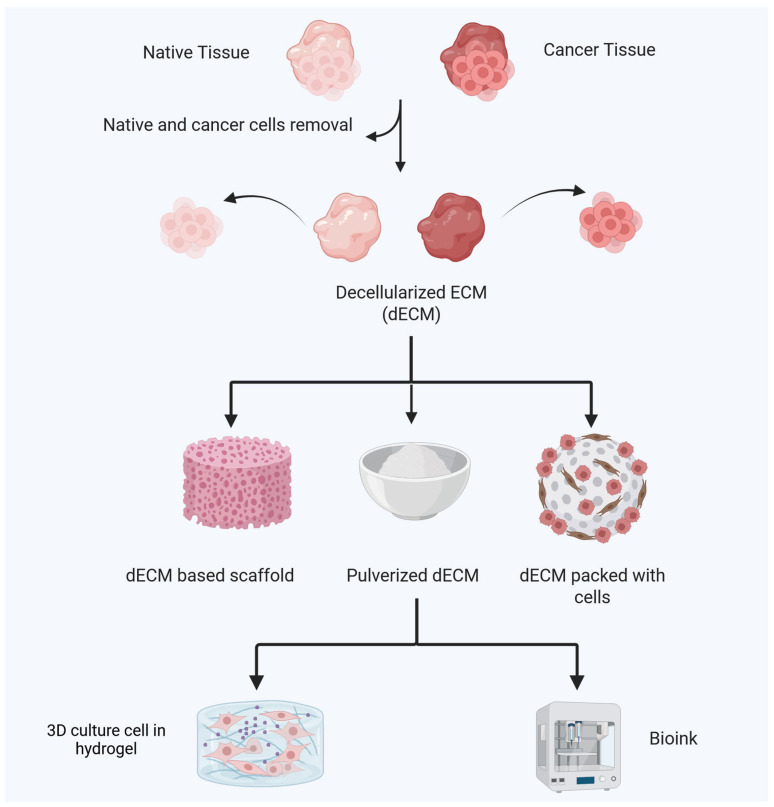
Recapitulating gastric tumor microenvironments using decellularized extracellular matrix (dECM): This schematic outlines the workflow for creating in vitro 3D models of gastric cancer using gastric tissue-derived dECM to study collagen dynamics in gastric cancer.

Then, decellularized gastric tissue provides a biomimetic 3D platform to study pathological collagen reorganization and crosslinking in gastric cancer. By preserving native ECM architecture, including region-specific collagen alignment and crosslinks altered in tumors, these scaffolds enable researchers to recapitulate the tumor microenvironment. When repopulated with cancer cells in 3D models, they reveal how collagen remodeling drives invasion and metastasis, offering a physiologically relevant tool to investigate ECM-targeted therapies (Figure 5).

## 6. Conclusions and Future Research Directions

Despite a decrease in the rate of gastric cancer cases, a population-based modeling study predicts a 62% increase in both annual new cases and deaths from gastric cancer by 2040 [1,2]. Although surgical therapy, chemotherapy, and immunotherapy have advanced, survival rates are still low because tumors are highly drug-resistant [186]. Given that the metastatic potential of gastric cancer strongly impacts the available therapies and patient survival, the focus of biomedical research on gastric cancer is centered on (1) the determination of new methods for the prevention and early diagnosis of gastric cancer, improving the staging and clinical significance of gastric cancer through molecular markers that complement morphological and histopathological methodologies; (2) investigation into molecular targets aimed at enhancing the effectiveness of existing treatments by focusing on the collagenous matrix while maintaining normal tissue homeostasis, especially in tissues characterized by high collagen turnover.

The influence of the tumor microenvironment in metastatic gastric cancer has been demonstrated at all phases of tumor growth [29,189]. It has been described that the stiffness of the ECM during tumoral progression can promote proliferation, drug resistance, and invasion of primary tumors [10,95,189]. This review has provided an in-depth analysis of how collagen within the TME becomes a key regulator of malignancy, operating through a complex interplay of biochemical and physical processes. Nevertheless, the mechanisms underlying collagen assembly and degradation mediated by PLOD, LOX, and MMP isoenzymes in the TME are still not fully elucidated. Furthermore, understanding the expression patterns of PLOD, LOX, and MMP in relation to tumor architecture may have valuable implications for predicting the severity of early-stage gastric cancer metastasis.

In addition, the clinical significance of this collagen-mediated immunosuppression is profoundly important. During the recruitment of tumor-related signals, various immune cell components infiltrate the immune microenvironment, engage closely with cancer cells, and subsequently interact with one another to collectively promote tumor development and also direct immunosuppressive signals [7,8,13,190]. Recent analyses of immunotherapy trials have revealed that high collagen density and particular collagen gene signatures serve as strong indicators of unfavorable responses to checkpoint inhibitors across various cancer types [191]. Consequently, approaches aimed at normalizing or reducing collagenous ECM have become a vital area of focus in addressing immunotherapy resistance. In this context, it is feasible to target biosynthesis, degradation, and signal transduction pathways to modulate collagen levels in the TME. In fact, studies demonstrate that the specific manipulation of collagen genes using gene-editing tools can offer durable or modifiable therapeutic solutions, with applications ranging from fibrotic diseases to hereditary connective tissue disorders. The specificity, reversibility, and personalized design of these interventions will be key to their future clinical application. Several recent studies have shown that gene editing using CRISPR-Cas9 offers a promising approach to treating fibrotic and connective tissue diseases, in which collagen plays a central role in pathophysiology. One of the most innovative approaches has been presented by Daliri et al. (2023) [192], who used a base editing system (ABE8e) to introduce a point mutation in the promoter of the COL1A1 gene in fibroblasts, significantly reducing the transcription and translation of type I collagen. This permanent modification, applied to the CAAT box of the promoter, showed efficacy without activating compensatory mechanisms, suggesting specific gene control over extracellular matrix production. This strategy has therapeutic implications in both chronic fibrosis and desmoplastic solid tumors, where excess collagen I promotes tumor progression. While the potential of permanent edits is recognized, the possibility of reversible strategies in physiological contexts is also discussed, in addition to the need for validation in more complex cellular and animal models.

The progression of gastric cancer is affected by the tumor microenvironment, underscoring the importance of establishing a biologically relevant environment for in vitro research. Three-dimensional biomimetic gastric cancer models utilizing decellularized extracellular matrix (dECM) represent a substantial advancement in oncological research, as they combine the structural fidelity of native ECM with the adaptability of in vitro approaches [173]. These models hold great potential for advancing our understanding of gastric cancer pathogenesis and improving therapeutic strategies. Additionally, patient-derived organoids seeded in dECM retain genetic and phenotypic heterogeneity, facilitating precision oncology approaches. These models have been used to evaluate chemotherapeutic responses, immunotherapy efficacy, and resistance mechanisms, offering a high-throughput platform for drug discovery.

Improving our understanding of stomach cancer cell responses requires the development of tissue-specific biomaterials and the control of matrix stiffness to mimic collagen assembly in the gastric microenvironment. In this sense, porcine-derived dECM offers distinct advantages, including structural homology to human gastric ECM, ethical procurement, and scalability [32].

Then, decellularized gastric tissue scaffolds provide a critical platform for investigating extracellular matrix remodeling in gastric cancer, particularly collagen reorganization and crosslinking within the tumor microenvironment. By removing cellular components while preserving native architecture, these scaffolds retain region-specific collagen fibril alignment, density, and biochemical cues altered in malignancy, such as increased linearization, lysyl oxidase (LOX)-mediated crosslinking, and pathological stiffening. When repopulated with gastric cancer cells or patient-derived organoids in 3D models, these scaffolds recapitulate TME-specific collagen dynamics. Tumor cells actively remodel the decellularized ECM and can reveal how crosslinking-driven matrix rigidity influences invasion, metastasis, and treatment resistance [186]. This approach enables targeted study of collagen-ECM crosstalk, allowing researchers to manipulate crosslinking enzymes (e.g., LOX inhibition) or stiffness parameters to dissect their roles in gastric cancer progression, offering a physiologically relevant alternative to synthetic or natural hydrogels for evaluating therapeutic strategies.

Bringing back the natural ECM features in the dECM scaffold creates a chance to build a tumor model that closely mimics real tumors for future studies. Despite their advantages, dECM-based models face limitations, including batch-to-batch variability, difficulty in scaling, and the need for standardized decellularization protocols. Future research should focus on optimizing biofabrication techniques, such as 3D bioprinting with dECM bioinks, to enhance reproducibility and complexity. Integrating microfluidics for dynamic nutrient and oxygen gradients could further improve physiological relevance [186].

With a deeper understanding of the interactions between cancer cells and their microenvironment, innovative anti-tumor strategies targeting collagen-producing cells are likely to be increasingly incorporated into clinical practice, supporting the advancement of new therapeutic options for patients with gastric cancer.

## Figures and Tables

**Table 2:** Comparative general features between dECM and Matrigel.

Feature	dECM	Matrigel
Source	Derived from decellularized native tissues (e.g., lung, heart, liver)	Basement membrane extract from Engelbreth–Holm–Swarm (EHS) mouse sarcoma
Composition	Contains native ECM proteins (collagens, proteoglycans, etc.) specific to the tissue of origin	Primarily composed of laminin, collagen IV, heparan sulfate proteoglycans, and entactin
3D Structure	Preserves the native 3D ultrastructure of the tissue, providing a more physiological environment	Forms a dense, non-fibrous gel with a reticular structure
Cellular interactions	Supports cell adhesion, migration, proliferation, and differentiation in a tissue-specific manner	Supports cell adhesion, spreading, and migration, primarily through interactions with integrins
Growth Factors	Contains growth factors present in the original tissue, which can be tissue-specific	Contains growth factors, but their concentrations and types can vary and may not be tissue-specific
Variability	Can exhibit batch-to-batch variability due to differences in donor tissues and decellularization protocols	Known for batch-to-batch variability due to its source
Cost	It can be more expensive than Matrigel, depending on the tissue source and decellularization method	Relatively inexpensive and readily available
Applications	Tissue engineering, regenerative medicine, organoid culture, drug screening, and disease modeling	Cell culture, 3D cell culture, angiogenesis assays, and in vitro studies

## Data Availability

Not applicable.
